# HIV prevalence, risky behaviors, and discrimination experiences among transgender women in Cambodia: descriptive findings from a national integrated biological and behavioral survey

**DOI:** 10.1186/s12914-017-0122-6

**Published:** 2017-05-23

**Authors:** Siyan Yi, Chanrith Ngin, Sovannary Tuot, Pheak Chhoun, Srean Chhim, Khuondyla Pal, Phalkun Mun, Gitau Mburu

**Affiliations:** 1 KHANA Center for Population Health Research, No. 33, Street 71, Tonle Bassac, Chamkar Mon, Phnom Penh, Cambodia; 2 Center for Global Health Research, Touro University California, Vallejo, USA; 3 FHI 360, Phnom Penh, Cambodia; 4 National Center for HIV/AIDS, Dermatology and STD (NCHADS), Phnom Penh, Cambodia; 5 Division of Health Research, Lancaster University, Lancaster, UK

**Keywords:** HIV, Sexually transmitted infections (STI), HIV prevention, Transgender women, Sexual behaviors, Cambodia

## Abstract

**Background:**

Transgender people are disproportionately affected by HIV. Despite their high vulnerability to HIV, lack of adequate epidemiological and surveillance data related to this population in many countries prevents provision of appropriate services. This paper summarizes descriptive findings from a national integrated biological and behavioral survey and discusses policy implications of the findings on HIV prevention among transgender women in Cambodia.

**Methods:**

This cross-sectional study was conducted between December 2015 and February 2016. Participants were recruited from 20 sites in the capital city and 12 provinces of Cambodia using Respondent Driven Sampling (RDS) method. Behavioral data were collected through structured questionnaire interviews, and rapid finger-prick HIV testing was performed. Descriptive data analyses were conducted using STATA.

**Results:**

This study included 1,375 transgender women with a mean age of 25.9 years (SD = 7.1). The overall prevalence of HIV was 5.9%. The prevalence of HIV was significantly higher among urban participants compared to their rural counterparts (6.5 vs. 2.6%, *p* = 0.02). Almost one in five (19.6%) had never been tested for HIV prior to the study. Overall, 45.0% reported ever using gender affirming hormones. More than one-third (39.1%) reported not using condoms in their last sex, 29.8% had engaged in sex in exchange for money/gifts, and 14.0% reported that they had experienced at least one symptom of sexually transmitted infections (STI) in the past year. About one in ten (10.1%) reported having used some form of amphetamine-type stimulant drugs, while 6.5% reported having sex during or after using illicit drugs. A significant number of participants experienced sexual abuse (39.2%), losing a job (24.3%), or physical abuse (23.6%) because of their transgender identity. In addition, 82.9 and 88.9% would be willing to use the HIV self-test and pre-exposure prophylaxis (PrEP), respectively, if they become available.

**Conclusions:**

The high prevalence of HIV, STI, and related risk behaviors among transgender women in Cambodia is of great concern, suggesting an urgent need to further expand tailored prevention interventions for this key population focusing on individual, social, and structural drivers of HIV. HIV self-test and PrEP should be explored as a priority.

## Background

Transgender people are disproportionately affected by HIV. The term “transgender” refers to a diverse range of people whose gender identities differ from the sex assigned to them at birth [1, 2]. A recent review by Baral et al. shows that transgender women have a pooled HIV prevalence of 19.1% [3], which is a 49-fold increased odds of HIV infection compared with non-transgender adults of reproductive age [2].

An increasing amount of epidemiological research has highlighted factors that increase HIV vulnerability among transgender people. Gender-based sexual violence that transgender people experience due to their gender identity [4] increases risk of HIV infection [5]. Stigma prevents access to HIV services and care [6], and is often associated with inconsistent condom use in anal intercourse and sex work among transgender women [7, 8]. Coinfection with perianal and other sexually transmitted infections (STI) could also facilitate acquisition and transmission of HIV [2, 9]. In addition, recent studies suggest ways in which gender affirming hormone use could impact HIV vulnerability [2]. Furthermore, sex work is often associated with higher prevalence of HIV among transgender people [10].

Whereas evidence showing the high vulnerability to HIV of this population is increasing, transgender people are generally underserved in national HIV responses [11]. For instance, the majority of countries do not include transgender in the national HIV plans and strategies [3]. This is often confounded by lack of adequate data related to transgender people in many countries [2]. Population-based representative studies and routine surveillance among transgender people are generally limited globally [12]. In addition, there is a paucity of data to guide clinical care of transgender people on antiretroviral and gender-affirming hormone therapies [13]. Furthermore, transgender people are often subsumed under men who have sex with men (MSM) in many countries [11, 14], which makes it difficult to provide tailored responses to their behaviors and vulnerabilities distinct from those of MSM [7, 11]. As a result, HIV programs are not effective for this population [7].

Research and dissemination of findings related to transgender people are required to inform policy and programming for these particularly vulnerable people. In this article, we present descriptive findings from a large-scale national integrated biological and behavioral survey among transgender women in Cambodia.

## Methods

### Study design, settings, and participants

The cross-sectional study was conducted between December 2015 and February 2016 in 13 major sites (1 capital city and 12 provinces) of Cambodia. Respondent Driven Sampling (RDS) method was used to recruit participants with support from community-based implementing partners of KHANA, the largest national NGO providing community-based HIV prevention, care, and support services in Cambodia [15]. Among the 13 study sites, participants were recruited in 20 specific locations (six locations in Phnom Penh and 14 locations in the provinces). The number of the selected locations was determined based on the proportion of the required sample size in each study site, and the estimated population of transgender women in each site. Eligible participants were individuals who: (1) were biologically male at birth and self-identified as a woman, (2) were aged 18 years or over, (3) reported having sex with at least one man in the past 12 months, (5) could speak Khmer, and (6) were able and willing to provide written consent to participate in the study.

Four initial seeds were identified at each site by outreach workers from KHANA’s implementing partners based on age (two seeds aged 18 to 24 and the other two seeds aged 25 or older). These seeds had to meet the eligibility criteria for participation and have an established and large social network comprising about 10 or more other transgender women in their given location. Eligibility to participate as a seed was determined by the leader of data collection team using a paper-based eligibility form. Each seed was given three coupons and asked to refer three additional transgender women. US$2 was given to each seed for a successful referral. Each seed was expected to extend to 3–6 “recruitment waves” in each site. If the initial seeds did not recruit participants or if enrollment was halted because all recruitment chains had “dried up” (i.e. stopped recruiting), additional seeds were selected based on the above criteria. In total, 80 seeds were selected by the implementing partners’ outreach workers initially.

### Data collection and training

Data were collected by two teams with eight personnel each that included one field supervisor, five interviewers, one lab technician, and one counselor from the Provincial AIDS and STI Program. The field supervisor conducted eligibility screening of the participants. Each consenting participant was assigned a unique personal identification number, which was used to link all data collected from each participant. The unique personal identification number was not linked with any personal data to protect confidentiality. The counsellor then explained objectives of the study in details, including the process of HIV testing, potential risks and benefits of participation, and obtained written informed consent. This was followed by HIV counselling conducted by the counsellor, and HIV testing conducted by the lab technician. Finger-prick-based HIV testing was conducted using Determine™ test, according to the national protocol [16]. HIV testing and counseling were performed in accordance with all applicable national guidelines.

After the HIV test was conducted, an interviewer administered the questionnaire in a private room using an Android tablet. At the end of the interview, the counsellor provided the HIV test result and post-test counselling. Participants with a reactive result were referred to the nearest HIV clinic for a confirmatory test, and were invited to come back to the implementing partner site for ongoing support and follow-up services. The field supervisor then provided a coupon incentive to the participants. Each participant received US$4 in cash to compensate for their time, and a package of three condoms. This amount of incentive was determined reasonable and equivalent to a typical meal for one person and the cost of transportation.

Prior to data collection, all interviewers and field supervisors were trained for three days on data collection methods and tool pretesting and reflection, to safeguard consistency, quality, and validity of the data. The training included interview techniques, confidentiality, and privacy, and provided opportunities for the study team the opportunity to rehearse questionnaire administration and other study procedures. During data collection, review sessions with interviewers were conducted regularly to review progress and communicate any problems or issues which required solving.

### Variables and measures

The questionnaire was developed using standardized and validated tools adapted from previous literature, the most recent Cambodia Demographic and Health Survey, and other studies among HIV key populations in Cambodia. It was initially developed in English and then translated into Khmer, the national language of Cambodia. Another translator then back-translated it into English to ensure that the “content and spirit” of every original item was maintained. Clear instructions and explanations were included to avoid any confusion during the interviews.

To design the study and develop the tools, consultative meetings were held with representatives of transgender women, community people, non-governmental organizations (NGOs), donor agencies, government officers, as well as researchers and practitioners working on HIV and AIDS in Cambodia. Prior to data collection, the questionnaire was pretested to ensure that the wording and contents of the questionnaire were culturally suitable, acceptable, and clearly understood by the study participants before it was finalized. In the pilot study, we conducted face-to-face interviews with 20 transgender women recruited from Phnom Penh to assess the contents, format, length, language, and appropriateness of the questionnaire. Necessary modifications were made based upon feedbacks from the pilot study and from the consultative meetings. The final version of the questionnaire was used for the main data collection.

The questionnaire collected the following information: (1) Socio-demographic characteristics; (2) transgender identity and related experiences; (3) sexual behaviors and condom use in different relationships; (4) accessibility to condoms and lubricants; (5) HIV/STI screening and care seeking behaviors; (6) substance use (alcohol, illicit drugs); and (7) experiences of stigma and discrimination in communities and health facilities.

### Data management and analyses

Data from the questionnaires collected via Android tablets were transferred onto a secure server after completion of each day of data collection. After synchronization, the data were cleared from the tablet. At the conclusion of the two-month data-collection, the data were downloaded from the secure server, tabulated, and transcribed into a Microsoft Excel format. Data were then imported into STATA (Version 12.0) for analyses. Prior to the analyses, data were weighted to account for the effect of RDS method. Descriptive analyses were conducted to calculate the counts and proportion (%) for categorical variables and means with standard deviations (SD) for continuous variables. Where applicable, Chi-square test or Student’s *t*-test was used to compare between groups.

### Ethical considerations

Participants were required to provide written consent after being informed in details about the study objectives, risk, and benefits. Participants were informed that they could withdraw from the study at any time. Interviews and HIV testing and counseling were conducted at private locations within drop-in centers, private houses, or offices of KHANA’s implementing partners, and confidentiality was ensured by assigning a personal identity number (PIN) to each participant and removing all personal identifiers. The study protocol was approved by the National Ethics Committee for Health Research (NECHR) of the Ministry of Health, Cambodia (No. 420 NECHR) and FHI 360’s Protection of Human Subjects Committee (PHSC No. 713897).

## Results

### Characteristics of the study participants

Characteristics of the study participants are shown in Table 1. Of the 1,375 participants, majority (83.4%) were urban dwelling, and 53.0% were younger than 25 years old. Mean age of the respondents was 25.9 years (SD = 7.1). Two-thirds of the participants (68.6%) completed high school, and 9.1% had higher education. Common occupations of the participants were hair dressers/beauticians (35.1%), laborers/farmers (17.5%), and entertainment workers (14.8%). More than one-third of the participants (38.6%) reported their average monthly income in the past six months of US$ 100–199, while 16.5% reported it to be more than US$ 300.

**Table 1:** Descriptive characteristics of the study participants

Variables	Number (%)
Community types	
Urban	1146 (83.4)
Rural	229 (16.6)
Age groups	
18– 24	729 (53.0)
25–34	503 (36.6)
35–44	99 (7.2)
≥ 45	44 (3.2)
Current marital status	
Married	7 (0.5)
Widowed/divorced/separated	18 (1.3)
Never married	1334 (97.2)
Refuse to answer	16 (1.2)
Formal education attained	
Primary (0–6 years)	307 (22.3)
High school (7–12 years)	943 (68.6)
Higher education (>12 years)	125 (9.1)
Main occupations	
Unemployed	64 (4.7)
Hair dresser/beautician	482 (35.1)
Office worker (government/private company staff)	50 (3.6)
Labor/farmer	241 (17.5)
Seller	149 (10.8)
Entertainment worker	203 (14.8)
Student	108 (7.9)
NGO staff	34 (2.5)
Other	44 (3.2)
Average monthly income in past six months (USD)	
< 100	351 (25.6)
100–199	530 (38.6)
200–299	266 (19.3)
≥ 300	226 (16.5)

Abbreviations: *NGO*, non-governmental organization; *USD*, United States dollar; *IQR*, interquartile range

### HIV prevalence

**Fig. 1:**
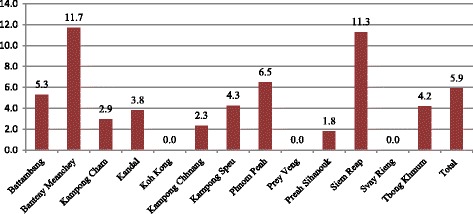
Prevalence of HIV among the study participants by study site

The overall prevalence of HIV was 5.9% (*n* = 81); of whom, 52% (*n* = 42) were not aware of their HIV status prior to the study. The highest prevalence of HIV was found among transgender women in Banteay Meanchey (11.7%) and Siem Reap (11.3%), followed by Phnom Penh (6.5%) and Battambang (5.3%). Similar HIV prevalence rates were found in Kampong Speu (4.3%), Tbong Khmum (4.2%), and Kandal (4.2%). No HIV-positive cases were detected in Koh Kong, Prey Veng, and Svay Rieng (Fig. 1). The prevalence of HIV was significantly higher among transgender women living in urban communities than among those living in rural communities (6.5 vs. 2.6%, *p* = 0.02). The highest prevalence of HIV was found among transgender women in the age group of 35–44 years old (13.1%), followed by those in the age group of >45 years old (11.4%). Transgender women in the age group of younger than 25 years old had the lowest HV prevalence rate at 3.0%.

### Gender identity and affirming hormone use

Table 2 shows that 42.2% of the participants identified themselves as female, while 57.2% identified as third gender. In the Cambodian context, “third gender” refers to transgender people (women or men). This term is now considered discriminating and unspecific; and it thus is not recommended for use in HIV or other related programs. About half of them (48.0%) reported dressing up as a woman all the time. Of total, 45.0% reported ever used hormone or other substances for beauty purposes such as pills (45.0%), injection (18.3%), and skin patches (14.0%). The injections were mostly reported to be performed by skilled personnel (67.8%), followed by non-skilled personnel (25.7%), and self-injection (6.5%). Of those who have injected the substances, 1.5% reported ever sharing needles while injecting the substances, and 9.2% had ever had an operation to change any part of their body to become more feminine.

**Table 2:** Gender identity and hormone use experiences of the study participants

Variables	Number (%)
Self-identified	
Female	580 (42.2)
Third gender	786 (57.2)
Uncertain	8 (0.6)
Frequency of dressing up as a woman	
All the time	660 (48.0)
Not all the time	715 (52.0)
Ever used hormone/non-hormone substances	618 (45.0)
Pill	561 (40.8)
Injection	252 (18.3)
Skin patches	192 (14.0)
How hormone/non-hormone substance hormone were injected	
Self-injection	17 (6.5)
Injected by skilled personnel	177 (67.8)
Injected by non-skilled personnel	67 (25.7)
Ever shared needles when injecting beauty substances	20 (3.2)
Ever had operation to change any parts of your body to become a woman	127 (9.2)

Abbreviations: *NGO*, non-governmental organization

### Sexual behaviors

As shown in Table 3, 8.5% reported ever had sex with a woman in their lifetime, and 2.2% reported ever had sex with a woman in the past 12 months. Of those who engaged in intercourse with a woman in the past 12 months, 66.7% reported having sex not in exchange for money, and 33.3% reported both in exchange and not in exchange for money.

**Table 3:** Sexual behaviors of the study participants with biological women and men

Sexual behaviors	Number (%)
Ever had sex with a woman (lifetime)	117 (8.5)
Ever had sex with a woman in the past 12 months	30 (25.6)
Type of female sexual partners in the past 12 months	
Female commercial partners	0 (0.0)
Female non-commercial partners	20 (66.7)
Both (commercial/non-commercial)	10 (33.3)
Role in anal sex with a man	
Insertive	29 (2.2)
Receptive	1145 (87.5)
Both	135 (10.3)
Had anal sex with a man, past 3 months	1183 (86.0)
Median number of male partners in past 3 months (IQR)	3 (1–7)
Used condom at last anal sex	732 (61.9)
Anal sex with male non-commercial partners in past 3 months	1122 (85.7)
Median number of male non-commercial partners in past 3 months (IQR)	3 (1–5)
Condom use in anal sex with male non-commercial partner in past 3 months	
Not always	697 (62.1)
Always	425 (37.9)
Reason for not using condoms with male non-commercial partner in the past 3 months	
We are in a relationship	438 (62.8)
He/she is not HIV/STI infected	216 (31.0)
Too high to use condom	37 (5.3)
No condom available	189 (27.1)
Feel better without condom	146 (20.9)
I am HIV-infected	2 (0.3)
I penetrated, so I am not at risk	4 (0.6)
Partner refused	119 (17.1)
Other	11 (1.6)
Had sex with a male commercial partner in the past 12 months	410 (29.8)
Median number of male commercial partners in the past 3 months (IQR)	3 (1–8)
Condom use with male commercial partners in the past 3 months	
Not always	164 (40.0)
Always	246 (60.0)
Reasons for not using condoms with male commercial partners	
We are in a relationship	59 (32.6)
She is not HIV/STI infected	25 (13.8)
Too high to use condom	17 (9.4)
No condom available	72 (39.8)
Feel better without condom	39 (21.6)
I penetrated, so I am not at risk	1 (0.6)
Partner refused	54 (29.8)
Other	7 (2.2)

Abbreviation: *HIV*, human immunodeficiency virus; *IQR*, interquartile range; *STI*, sexually transmitted infection

Table 3 shows information about participants’ sexual experiences with men. Nearly all respondents (97.9%) have had sexual intercourse with a man. The majority (87.5%) reported a receptive role, while only 2.2% reported an insertive role, and 10.3% reported both roles. In the past three months, 86.0% had anal sex with a man, of which 61.9% used condom in their last sex. Among all participants, 81.6% had sex not in exchange for money/gifts, of which 62.1% reported not always using condoms in the past three months by giving the reasons that they were in relationship (62.8%); their partners were not HIV/STI infected (31.0%); no condom was available (27.1%); they felt better without condom (20.9%); and their partners refused to use it (17.1%). Among all participants, 29.8% had sex in exchange for money/gifts, of which 40.0% reported not always using condoms in the past three months by giving the reasons that no condom was available (39.8%); they were in relationship (32.6%); their partners refused to use it (29.8%); and they felt better without condom (21.6%).

### Access to condoms and lubricants

**Fig. 2:**
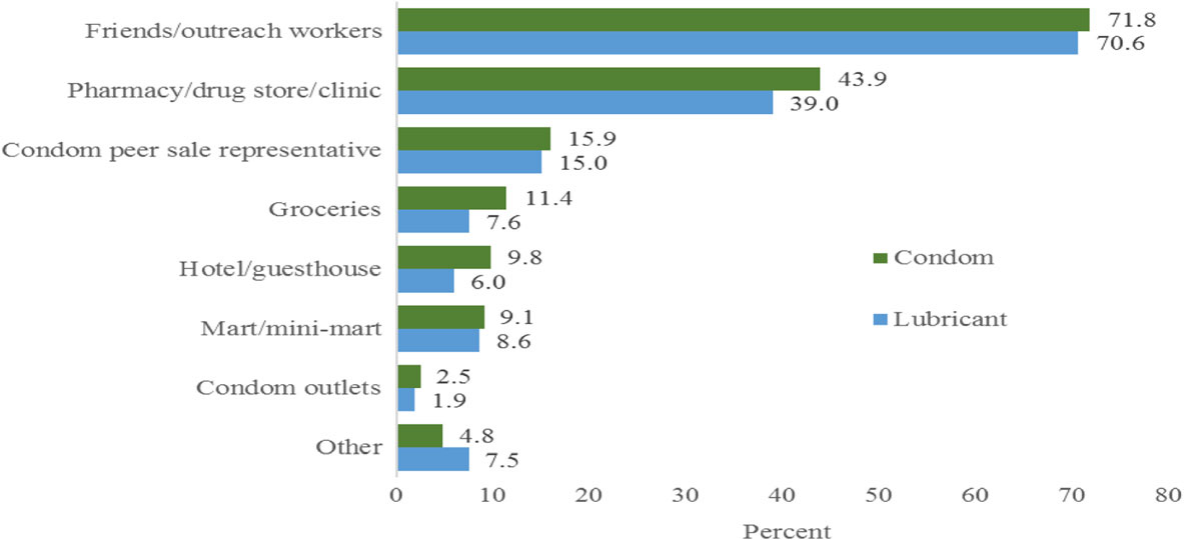
Access to condoms and lubricant by type of facility in the past 12 months

Figure 2 shows the access to condoms and lubricant in the past 12 months. The most commonly reported sources of condoms and lubricant were friends/outreach workers (71.8 and 70.6% for condoms and lubricant, respectively), followed by pharmacies/drug stores/clinics (43.9 and 39.0% for condoms and lubricant, respectively).

### STI symptoms and treatment

Table 4 shows the experiences of STI symptoms and treatment seeking behaviors among transgender women in this study. Of the total survey sample, 14.0% reported that they had experienced at least one STI symptom in the past 12 months. The most commonly reported symptoms were symptoms on the anus (6.1%). Of those with the symptoms, 138 (71.1%) reported seeking treatment for their most recent symptom from an NGO facility (21.5%), private pharmacy (16.4%), public facility (16.4%), and private facility (14.9%).

**Table 4:** STI symptoms and treatment experiences of the study participants

Variables	Number (%)
STI symptoms experienced in the past 12 months	193 (14.0)
Cut or sores in the genital area	46 (3.4)
Swelling in the genital area	16 (1.2)
Abnormal urethral discharge	36 (2.6)
Symptoms on the anus	84 (6.1)
Symptom in the mouth/throat	39 (2.8)
Facility where treatment for the most recent STI symptoms was received	
Did not seek treatment	57 (29.2)
Private pharmacy	32 (16.4)
Private facility	29 (14.9)
Public facility	32 (16.4)
NGO facility	42 (21.5)
Traditional healer	3 (1.5)

Abbreviations: *NGO*, non-governmental organization; *STI*, sexually transmitted infection

### HIV testing, treatment, and willingness to use PrEP

Table 5 shows that 19.6% had never been tested for HIV before, while 44.3% had been tested within the past 6 months. Of those who had been tested and received results, 3.6% reported being HIV positive. Among those who reported being HIV positive, 94.9% were currently on ART. Majority of the testing (69.7%) were done at an NGO facility.

**Table 5:** HIV testing experience, status awareness, and willingness to use PrEP

Variables	Number (%)
Ever been tested for HIV	
Never	269 (19.6)
1–3 months	431 (31.4)
4–6 months	178 (12.9)
7–12 months	346 (25.1)
> 12 months ago	151 (11.0)
Received results from the last HIV test	1079 (97.5)
Currently on ART	37 (94.9)
Place of the most recent HIV test	
Private facilities	119 (10.8)
Public facilities	208 (18.8)
NGO facilities/community based HIV testing	771 (69.7)
Other	9 (0.8)
Willingness to use HIV self-test if it were available	842 (82.9)
Ever heard of Pre-exposure prophylaxis (PrEP)	359 (37.6)
Likeliness of using PrEP	
Unlikely	79 (8.3)
Likely	849 (88.9)
Not sure	27 (2.8)
Preferred place to access PrEP if available	
Local NGOs	511 (53.5)
ART clinic	148 (15.5)
Pharmacy	270 (28.3)
Other	26 (2.7)

Abbreviations: *ART*, antiretroviral therapy; *HIV*, human immunodeficiency virus; *NGO*, non-governmental organization; *PrEP*, pre-exposure prophylaxis

Many participants (82.9%) said they were willing to use the HIV self-test if it were available. When asked if they had ever heard of pre-exposure prophylaxis (PrEP), 37.6% participants answered ‘Yes.’ Regarding the likeliness of using PrEP, 88.9% said they would likely use it and that they would prefer to access the PrEP at a local NGO (53.5%) over other locations (Table 5).

### Substance use

Table 6 depicts the reported alcohol and illicit drug use among transgender women in this study. Of total, 75.9% reported drinking at least one can of beer or a glass of wine in the past 3 months. More than half (56.1%) responded that this occurred less than once a month, while 8.1% responded four or more times a week. Of total, 10.1% reported having used some form of amphetamine-type stimulants (Yama, Crystal Ice, Ecstasy), while 0.9% reported having used other drugs (marijuana, heroin, etc.). Of total, 1.5% reported having injected some form of illicit drugs in the past 3 months, and 6.5% reported having sex during or after using illicit drugs.

**Table 6:** Substance use among the study participants

Variables	Number (%)
Drank at least one can of beer or glass of wine in the past 3 months	1042 (75.9)
Frequency of having more than 5 drinks in one day in the past 3 months	
Never more than five drinks	336 (24.4)
Less than once a month	771 (56.1)
1–3 times a week	157 (11.4)
4 or more times a week	111 (8.1)
Ever used illicit drugs in the past 12 months	
Never	1224 (89.0)
Yes, ATS (Yama, crystal ice, ecstasy)	139 (10.1)
Other drugs (marijuana, heroin, etc.)	12 (0.9)
Ever injected any illicit drugs in the past 3 months	20 (1.5)
Ever had sex during/after using illicit drugs in the past 3 months	89 (6.5)

Abbreviation: *ATS*, Amphetamine-type stimulants

### Discrimination experiences

**Fig. 3:**
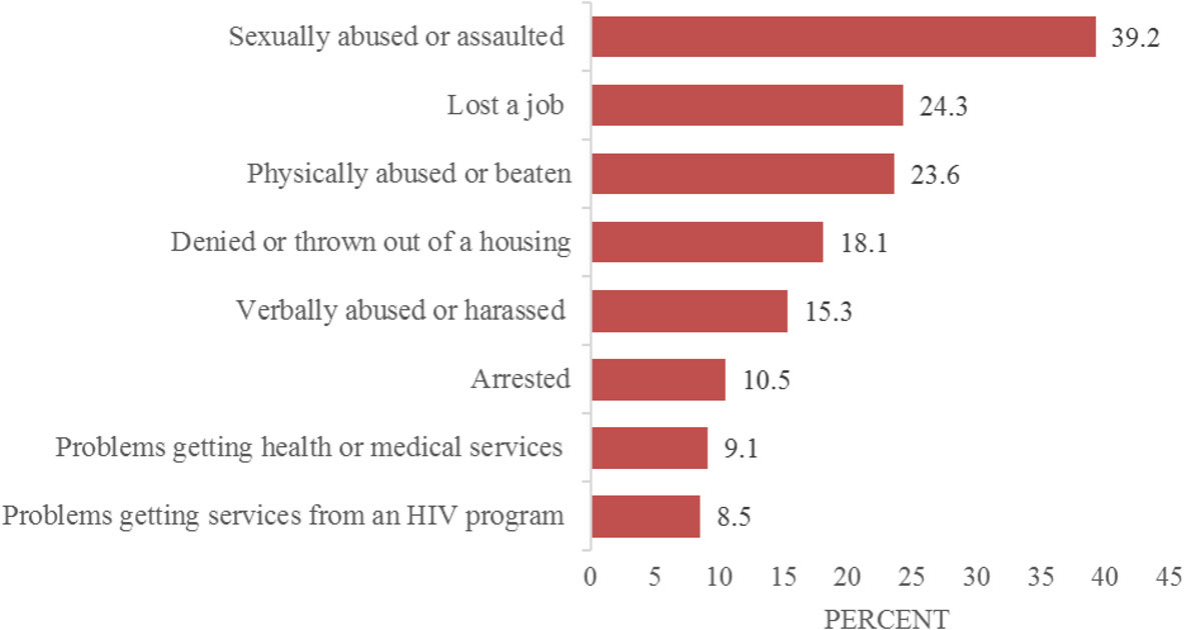
Discrimination experiences of the study participants

Figure 3 shows the discrimination experienced by transgender women in this study because of their transgender identity or gender presentation. Of total, 42.1% experienced problems getting a job, whereas 24.3% experienced problems losing a job. The next most common discrimination experienced was sexual abuse or assault (39.2%), while the least common form of discrimination reported in this study were problems getting services from an HIV prevention program (8.5%) and had problems getting health or medical services (9.1%).

## Discussion

There is growing interest in understanding the epidemiology of HIV among transgender people. This study set out to explore demographic, behavioral and health-related characteristics of transgender women in Cambodia. Results showed that the prevalence of HIV was 5.9% among the sample. In addition, the results provide useful data on high-risk sexual behaviors, STI symptoms, substance use, HIV testing, discrimination experiences, and willingness to use PrEP among this population.

The HIV prevalence found in our study is slightly higher than the 4.2% found by a recent study by Weissman et al. [17], but both are significantly higher than an earlier study conducted in 2010 which found a prevalence of 2.6% among transgender women [18]. However, direct comparison of findings from this study with those in the previous ones is difficult because of the differences in characteristics of the samples and measures. Our study was conducted in 13 sites (one city and 12 provinces), while the study by Weissman et al. was conducted in six sites [17] and the Bros Khmer study in seven sites [18]. The timeframes used to measure key variables, such as HIV testing and condom use, were also not consistent. Studies from Cambodia have consistently reported higher levels of HIV prevalence among transgender people compared to MSM [17, 18], underscoring the importance of separating transgender people from MSM so as to have an accurate picture of HIV epidemic among this group.

Since 2013, transgender women have been recognized as a separate population from MSM within the Standard Operating Procedures of Boosted Continuum of Prevention, Care and Treatment [19]. However, even within transgender people, differences were noted in HIV prevalence based on residence. The prevalence was significantly higher among transgender women in urban communities compared to those in rural areas, which suggests the need to better focus on cities and urban areas. These findings also buttress calls for geographic localization of HIV prevention efforts [10], given that HIV rates were much higher in Banteay Meanchey, Siem Reap, Phnom Penh, and Battambang provinces compared to other provinces.

An important implication from the study was related to combination prevention. The majority of the participants were sexually active, with 97.9% reporting having intercourse with men, mostly anal sex. However, a significant number of these did not use condoms consistently. Given the high prevalence of HIV in this group, these findings suggest that expansion of HIV prevention interventions for this group is warranted. This is particularly relevant given that two thirds of the participants had not heard of PrEP, but 88.9% were willing to use it. Combined with the high proportion (94.9%) of HIV positive participants who were on ART, these findings suggest that provision of PrEP could reduce incidence of HIV among this population. The high prevalence of sex work in exchange of money among this sample provides another reason to consider PrEP for transgender sex workers. Other studies have highlighted the heightened vulnerability and the needs for better tailored interventions for transgender sex workers [20, 21].

The low rate of HIV testing observed among transgender women in this study is of concern and suggests that significant effort is required to increase HIV testing uptake. In particular, HIV education and benefit of HIV testing should be widely promoted among this population. The lack of HIV awareness could influence HIV sexual behaviors, including the use of condoms, or commercial sex [22]. In addition, HIV testing is the first step towards early access to treatment, which also has high preventive benefits. Results from this study indicate that participants were willing to consider HIV self-testing, which could provide additional flexibility in access to testing. HIV self-testing has potential to reach individuals who are least likely to seek testing in a public clinic, hospital, voluntary confidential counseling and testing center (VCCT), or with a community-based non-governmental organization. Results from other settings indicate that self-testing may be acceptable to transgender people [23] and should be made available while considering issues related to confirmation and linkage to care, cost [24], quality assurance, as well as support needs of different potential users of self-tests [25].

Our study indicates that 6.5% of the participants reported having sex during or after using illicit drugs, so called “Chem sex”, which may increase sexual risk taking [26, 27]. Furthermore, 45% of the participants had ever used gender affirming hormones, which included through injections in 18% of the cases. Our results also indicate that the injecting practices were often unsafe (by self-injection or non-skilled providers) and included sharing of needles. Therefore, HIV prevention efforts should explore provision of PrEP and education regarding the use of drugs during sex, while considering how safe needle exchange interventions can be integrated for those who inject drugs or hormones.

Our study shows the importance of community-based outreach services in increasing access to HIV prevention services. Peers and outreach workers were the sources of condoms for 70% of the participants. In addition, findings related to STI suggested that the majority of the participants that experienced STI symptoms shunned public facilities as a source of treatment, with the majority seeking health care from NGO or private facilities and pharmacies. Furthermore, the majority of participants accessed HIV testing from community-based NGOs. These preferences may be related to the demand creation, peer-support building or less stigmatizing nature of community-based organizations and services in the study context. Evidence from other contexts indicate that when services, often community-based, are non-stigmatizing, gender-affirming, trans-specific and leveraged on local peers and resources, transgender people access them [28–30].

Conversely, these findings highlight a need to better understand how the capacity of public health facilities to provide friendly services to transgender people can be strengthened. In 2014, the World Health Organization released a global guidance on HIV programming for key populations, including transgender people [31]. More recently, implementation tools developed to assist the provision of comprehensive community-led responses for transgender people have been launched [32]. In Cambodia, these guidelines and implementation tools need to be adapted and implemented.

Stigma and discrimination are powerful drivers of inequitable access to HIV and health services among transgender [6, 33] and other [34] populations. Yet HIV cannot be eliminated unless transgender persons can have universal access to HIV and health services. Our study shows that a significant number of transgender women experienced discrimination based on their transgender identity, including problems getting services from an HIV prevention program (8.5%) and other health and medical services (9.1%). In a similar Cambodian study, 54.8% of participants reported having experienced discrimination related to transgender identity in their lifetime [17]. Consequently, significant effort will be required to sensitize, and train health care providers so that they provide appropriate care with non-judgmental attitudes to transgender women. General practitioners, nurses and non-clinical health professionals working in public hospitals and HIV clinics need to be equipped with appropriate clinical skills including clerking, counselling, and examination skills so as to provide appropriate care to transgender women. This is particularly important because 39.2% of the participants reported experiencing sexual abuse or assault, which can increase HIV vulnerability and requires access to health and medical services.

More broadly, the intersectionality of multiple deleterious determinants of health, including drug use or abuse, violence or stigmatization, poor mental health, internalized transphobia, and economic hardships may potentiate HIV vulnerability [35], reduce uptake of services [34], and contribute to poor HIV treatment outcomes [2] among different populations, including transgender people. Hence, multi-sectoral interventions to mitigate these complex interacting syndemic factors in an integrated fashion are required. Importantly, this approach should include sectors beyond physical health, such as mental health, legal, policing, and social protection sectors, to mitigate arbitrary arrests [6, 36], lack of employment [37], criminalization, victimization [38], violence [5, 38], and other structural drivers of vulnerability to HIV among this population.

Findings from this study should be cautiously interpreted in light of the design, recruitment, methods of data collection, and sample profile. As this was a cross-sectional study, it captures a snapshot view of the population studied, and may not document changes in measured variables over time. Our study sample had relatively educated participants, which may not be representative of all transgender women in the country, and were initially recruited from those who had some contact with outreach workers. Future studies should be more cautious about selecting seeds that are more representative of sub-populations of transgender women by considering their socio-demographic characteristics such as education levels, occupations, incomes, etc. Sexual behaviors and other sensitive information were collected from the participants through a self-reported questionnaire, which is often at high risk of bias [39]. Despite these limitations, this paper provides important information that can be built upon to inform programming and policy.

## Conclusions

There is a growing interest in understanding the epidemiology of HIV among transgender people. This article presents findings from a cross-sectional study among transgender women in Cambodia, showing the high HIV prevalence and risky sexual behaviors among the participants. These findings call for an urgent need to further expand and refine prevention interventions for transgender women, focusing on individual, social, and structural domains of vulnerability. This study therefore should inform policy and programmatic interventions to promote health and prevent the HIV vulnerability among transgender people in Cambodia.

## Abbreviations

AIDS: Acquired immune deficiency syndrome
ART: Antiretroviral therapy
HIV: Human immunodeficiency virus
IQR: Interquartile range
MSM: Men who have sex with men
NGO: Non-governmental organization
PIN: Personal identification number
PrEP: Pre-exposure prophylaxis
RDS: Respondent driven sampling
SD: Standard deviation
STI: Sexually transmitted infections
TG: Transgender
USD: United States dollar
VCCT: Voluntary confidential counseling and testing center
